# Isolation, Culture and Functional Characterization of Glia and Endothelial Cells From Adult Pig Brain

**DOI:** 10.3389/fncel.2019.00333

**Published:** 2019-07-23

**Authors:** Goutam Kumar Tanti, Rajneesh Srivastava, Sudhakar Reddy Kalluri, Carina Nowak, Bernhard Hemmer

**Affiliations:** ^1^Department of Neurology, School of Medicine, Technical University of Munich, Munich, Germany; ^2^Munich Cluster for Systems Neurology, Munich, Germany

**Keywords:** glial cells, endothelial cells, reliable method, pig brain, microglia, astrocytes, oligodendrocytes

## Abstract

Primary cultures of glial and endothelial cells are important tools for basic and translational neuroscience research. Primary cell cultures are usually generated from rodent brain although considerable differences exist between human and rodent glia and endothelial cells. Because many translational research projects aim to identify mechanisms that eventually lead to diagnostic and therapeutic approaches to target human diseases, glia, and endothelial cultures are needed that better reflect the human central nervous system (CNS). Pig brain is easily accessible and, in many aspects, close to the human brain. We established an easy and cost-effective method to isolate and culture different primary glial and endothelial cells from adult pig brain. Oligodendrocyte, microglia, astrocyte, and endothelial primary cell cultures were generated from the same brain tissue and grown for up to 8 weeks. Primary cells showed lineage-specific morphology and expressed specific markers with a purity ranging from 60 to 95%. Cultured oligodendrocytes myelinated neurons and microglia secreted tumor necrosis factor alpha when induced with lipopolysaccharide. Endothelial cells showed typical tube formation when grown on Matrigel. Astrocytes enhanced survival of co-cultured neurons and were killed by Aquaporin-4 antibody positive sera from patients with Neuromyelitis optica. In summary, we established a new method for primary oligodendrocyte, microglia, endothelial and astrocyte cell cultures from pig brain that provide a tool for translational research on human CNS diseases.

## Introduction

Glial cells are essential to maintain hemostasis, protect neurons and form myelin in the central nervous system (CNS) (Abbott et al., 2006; Bayraktar et al., 2014; Nave and Werner, 2014; Zou et al., 2014; Domingues et al., 2016; Mosser et al., 2017). They make up more than 50% of all CNS cells (von Bartheld et al., 2016). The different glial cells include (i) microglia, the brain resident macrophages, mononuclear phagocytes that are essential for immune responses in the CNS (Li and Barres, 2018), (ii) oligodendrocytes, which myelinate and provide support to the neurons, and (iii) astrocytes, which are important for the nutrition of neurons, hemostasis in the extracellular space and repair mechanisms. Apart from glial cells and neurons, the brain contains different types of connecting cells including endothelial cells and pericytes, which play a critical role in the formation and maintenance of the blood-brain barrier (Daneman and Prat, 2015; Obermeier et al., 2016). Many human CNS diseases are caused by dysfunction of glia (Bradl and Lassmann, 2010; Molofsky et al., 2012; Domingues et al., 2016; Hoffmann et al., 2018; Li and Barres, 2018) or endothelial cells (Zhao et al., 2015). Glia and endothelial cell cultures have provided an experimental setup to study their function and dysfunction *in vitro*. Most isolation and culture protocols so far are based on embryonic fetal brain from rodents. However, it has become more and more evident that substantial differences exist between human and rodent glia (Oberheim et al., 2009; Semple et al., 2013; Zhang et al., 2016). Likewise, human astrocytes are morphologically and functionally quite distinct from rodent astrocytes. The kinetics of myelination by oligodendrocytes or the aging of microglia is substantially different between rodents and humans (Chandran et al., 2004; Galatro et al., 2017). Also, the expression profile of genes and proteins in human and rodent CNS is quite distinct (Zhang et al., 2016; Galatro et al., 2017). Given these constraints of rodent glia, primary glia cultures from humans or closely related species are considered to be more relevant for investigating human diseases and their treatment. Because the access to human brain is very limited other species that are closer to human have to be considered for establishing primary glia cultures. Pig is quite close to human in terms of similarity of organ functioning, anatomical localization, and gene pool sharing (Swindle et al., 2012; Yu et al., 2015). Pig brain tissue is widely and easily available without the need to kill animals only for research purposes.

We developed a reliable method to separate microglia, oligodendrocytes, endothelial cells and astrocytes from adult pig brain for primary cultures. The glia and endothelial culture were kept for up to 8 weeks without significant change in their properties. Functional integrity of the cell cultures was demonstrated by different bioassays.

## Materials and Methods

### Isolation and Separation of Specific Cell Types

#### Dissociation of Pig Brain to Make Cell Suspension

1. Pig brain from 2 to 3 months old animal was obtained from the slaughter house, stored on ice until arrival in the laboratory. The transfer time was between 1 and 2 h. Upon arrival in the laboratory the brain was put in cold Phosphate Buffer Saline (PBS) containing 1% Fetal Calf Serum (FCS) and 1% antibiotic cocktail of Penicillin and Streptomycin (Pen/Strep). We used pig brain from animals, which were slaughtered for meat production. No animal was killed for the study. Thus no approval by animal protection authority or an ethic board was necessary.
2. Brain tissue were dissociated in Hank’s Balanced Salt Solution (HBSS) containing DNAse I and Hyaluronidase (the total ingredients are shown in Supplementary Table 1), generally, 10–15 g of tissues are digested in 10 ml of digestion buffer at 37°C in the water bath for 2 h. We used one hemisphere of the whole brain for cell isolation.
3. The tissue parts were passed through the varying size of pipettes for several times to enhance the formation of the cell suspension. Then the cell suspension was passed through the cells strainer of varying size (100 μm and 70 μm) from Falcon, Corning (Catalog Numbers 352350 and 352360).

Steps 1–3 take 2–3 h.

#### Myelin Removal From Cell Suspension

4. To every 12 ml of cell suspension, 4 ml of isotonic Percoll was added in a 50 ml falcon tube and mixed properly. 25 ml of DMEM/isotonic Percoll mixture (5:1 ratio) was added very carefully on top of the cell suspension to allow formation of a clear and visible gradient.
5. Cell suspension was then centrifuged in a swinging bucket rotor at 4,000 g for 75 min at 22°C without a brake. After centrifugation two layers formed. Cells remain in the layer just above the pellet. The topmost white layer is of myelin. The myelin was discarded carefully without disturbing the bottom layer.
6. Cells were carefully taken out from the lower layer and transferred to a new falcon tube. DMEM, twice the volume of the cell suspension, was added and the tube centrifuged at 2,500 g for 5 min to pellet down the cells. Cells were counted.
  Generally, we obtain ∼3.5 × 10^6^ cells per gram pig brain tissues with a viability of ≥ 90% of cells.
7. 1X Basic Defined Media (BDM, see Supplementary Table 2 for composition) was added to the cells to a total volume of 100 ml. The cells were incubated at 37°C in 5% CO_2_ for 30 min.

Steps 4–7 take 2–3 h.

#### Separation of Glia Cells

Different cell types were separated by lineage-specific labeling with antibodies and magnetic field based separation (specific reagents are displayed in Supplementary Table 3).

8. In general, ∼2 × 10^7^ cells were suspended in 160 μl of cold separation buffer [PBS containing 1% Bovine Serum Albumin (BSA)], then 20 μl of FcRn blocker and 20 μl of magnetically labeled CD11b was added. The cells were mixed carefully and incubated for 15 min on ice.
9. At the very end of the incubation, the maxi column from MACS was attached to a stand with strong magnetic field with pre-separation filter and was equilibrated with 10 ml of separation buffer. The unbound antibodies were washed off with 10 ml of separation buffer followed by centrifugation at 2,500 × *g*. Then 5 ml of separation buffer was added to the cells and the cells were passed through the maxi column, which was kept under a strong magnetic field. The labeled cells were retained and un-labeled cells were washed off. These flow through fraction of cells was collected and centrifuged. To increase stringency in the protocol, the column was washed with at least 10 ml of separation buffer. Then the column was taken out of the magnetic field and the labeled cells were eluted by passing of 5 ml of separation buffer with force in one go. The eluted cells were centrifuged and collected and kept aside.
10. The flow through fraction of cells were centrifuged and re-suspended in 5 ml of separation buffer.
11. Step 9 was repeated. The flow through fraction of cells was collected and marked as (CD11b−).
12. The eluted cells from step 9 and 11 were mixed and centrifuged. The eluted cells were re-suspended in 5 ml of separation buffer. The re-suspended cells were passed through fresh maxi column (optional). Step 9 was repeated. The flow through fraction from the second elution was discarded. The labeled cells were eluted and marked as CD11b+.
13. The flow through fraction from step 11 (called CD11b− cells) were centrifuged and re-suspended in 180 μl separation buffer. Twenty microliter of magnetically labeled O4 antibody was added to it and incubated for 15 min on ice.
14. Same as Step 9.
15. Same as step 10.
16. Same as step 11. The flow through fraction was collected and marked as (CD11b−, O4−).
17. The eluted cells from step 14 and 16 were mixed and centrifuged. The eluted cells were re-suspended in 5 ml of separation buffer. The re-suspended cells were passed through fresh maxi column. Step 9 was repeated. The flow through fraction from the second elution was discarded. The labeled cells were eluted and marked as O4+.
18. The flow through fraction of cells from step 15 (known as CD11b−, O4−) were centrifuged and suspended in 180 μl separation buffer. Twenty microliter of magnetically labeled CD31 antibody (for separating endothelial cells) was added to it and incubated for 15 min on ice.
19. Same as Step 9.
20. Same as step 10.
21. Same as step 11. The flow through fraction of cells was collected and marked as (CD11b−, O4−, and CD31−); these cells were used to culture astrocytes and neurons in different methods as mentioned below.
22. The eluted cells from step 19 and 21 were mixed and centrifuged. The eluted cells were re-suspended in 5 ml of separation buffer. The re-suspended cells were passed through fresh maxi column. Step 9 was repeated. The flow through fraction from the second elution was discarded. The labeled cells were eluted and marked as CD31+.

Steps 8–22 take 2–3 h.

### Cell Culture

All separated cell fractions were cultured in similar conditions but in different cell type specific media. The surface of the coverslips, plates, or flasks were coated with L-ornithine and laminin.

#### Preparation of the Plates/Flasks

1. The coverslips were kept in 100% HCl for 24–48 h and then kept in 100 ethanol for 2 weeks, ethanol was changed every 2/3 days.
2. The coverslips were inserted inside the well of a 24 well plate. Around 500 μl of 70% ethanol was poured in each well. The plate was kept in RT for overnight. Next day, the ethanol was aspirated and the plate was kept inside the culture hood under the UV for 2/3 h to sterilize and dry the plate.
3. Poly-L-ornithine was prepared. Generally, 20 mg/ml of Poly-L-ornithine is prepared in water and aliquoted, stored at −20°C. The final concentration was 20 μm/ml. 1 ml of the Poly-L-ornithine was used for 6 well plates whereas 500 μl and 100 μl of the same solution were used for 24 well and 96 well plates, respectively. Plates were kept in an incubator for at least 1 h at 37°C or overnight at 4°C.
4. Laminin was thawed, aliquoted and stored at −20°C. Laminin was prepared at 1 μg/ml in filtered distilled water and added to the plates after removing the Poly-L-ornithine. The plates were incubated for at least 1 h in the incubator at 37°C or overnight at 4°C.

Fluid was aspirated and the plates were washed once with PBS containing Calcium and Magnesium before usage.

#### Culturing Cells

5. Glial and endothelial cells were re-suspended in 1X BDM containing 10% FCS and 1X pen/strep. Serum was used to increase the adherence of cells to the surface of the culture plates and coverslips. Usually 24 well plates were used for culture for staining experiment. 2–3 × 10^4^ microglia, 3–4 × 10^5^, oligodendrocytes or astrocytes, 1 × 10^4^ endothelial cells were plated per well in 24 well plate.
6. After 12 h, BDM medium was exchanged by specific media to promote the growth of the different cell types (see Supplementary Table 4 for microglial, Supplementary Table 5 for oligodendrocytic, Supplementary Table 6 for endothelial and Supplementary Table 7 for astrocytic media composition). For culturing microglia, FCS is mainly used to enhance the survival and growth of cells (Collins and Bohlen, 2018). For maintaining the culture, we added FCS to 1X basal defined media (BDM). However, all functional experiments, e.g., microglia activation, were carried out with defined media (1X BDM) without FCS supplementation.
7. Neuronal culture required removal of the dominant astrocyte population from the triple negative cell fraction (CD11b−, O4−, CD31−, see separation of cells, step 20). To remove astrocytes, cells were cultured in the neuronal media (Supplementary Table 8) and treated with AraC for 24 h to kill proliferative cells. AraC was removed from the culture by washing the cells once with neuronal media and cells were further kept in neuronal media, which was replenished every second day with fresh neuronal culture media.

#### Flow Cytometric Analysis

Thirty thousand cells per sample were washed in FACS buffer (PBS containing 0.5% FCS) and fixed with 100 μl of 1X BD Cytofix/Cytoperm solution (cat #554722, BD Bioscience) for 20 min at 4°C. After two washes with 1X BD Perm/Wash^TM^ buffer, the cells were permeabilized with 100 μl of the same wash buffer for 20 min in the dark at room temperature. The cells were then washed twice with BD Perm/Wash^TM^ buffer. Primary antibodies diluted in BD Perm/Wash^TM^ buffer was added to the cells and incubated on ice for 1 h (see Supplementary Table 10 for different conditions and antibody dilutions used). Cells were washed twice with BD Perm/Wash^TM^ buffer. Secondary antibodies (with Alexa 647 IgG, 1:100) diluted in BD Perm/Wash^TM^ buffer was added and incubated for 25 min on ice. After two washes with BD Perm/Wash^TM^ buffer, 175 μl of FACS buffer added to the cells and analysis was carried out on a Beckman Coulter Cytoflex S flow cytometer.

#### Real-Time PCR

RNA was extracted from both freshly isolated and cultured cells. cDNA was synthesized from 2 μg of RNA in 20 μl of reaction using SuperScript^TM^ VILOfollowing manufacturer’s Master Mix from Thermo Fisher Scientific (cat #11754050) following manufacturer’s protocol. qPCR was carried out for different genes with TaqMan probe procured from Thermo Fisher Scientific (see Supplementary Table 9).

#### Immunocytochemistry

For immunostaining with a single antibody, the cells were washed once with 1X PBS for 5 min each and fixed with 3.7% formaldehyde in 1X BDM containing 10% FCS for 15 min and washed twice with 1X PBS for 10 min each. Cells were then permeabilized and blocked with buffer containing 10% goat serum (or serum from species where secondary antibody is raised), 0.2% Triton X-100 and FcRn blocker (at a dilution of 1 to 10) in 1X PBS for 2 h at room temperature. The cells were washed twice with 1X PBS for 5 min each. Coverslips with cells were then incubated with primary antibody (see Supplementary Table 10 for different conditions and antibody dilutions used) overnight at 4°C. Then the cells were washed three times with 1X PBS for 5 min each. The coverslips were then incubated in secondary antibody for 1 h at 1:500 dilution in 1X PBST (PBS containing 0.1% Tween 20). Coverslips were then washed three times with 1X PBS for 10 min each, mounted on slides with ProLong^TM^ Diamond Antifade Mountant containing DAPI from Thermo Fisher Scientific (cat #P36961). For double immunostaining, the cells were incubated with blocking buffer containing 10% goat serum (or serum from species where secondary antibody is raised), 0.2% triton-X 100, FcRn blocker (at a dilution of 1 to 10), and 4 drops per ml of avidin from ABC kit of vector lab for 2 h and then washed three times with 1X PBS for 5 min each. Cells were incubated overnight in primary antibody in PBST containing 4 drops per ml of biotin. Cells were washed three times with 1X PBS for 10 min each. Secondary antibody corresponds to particular primary antibody conjugated with biotin was added at a dilution of 1 to 500 in PBST and incubated for 1 h. Cells were washed three times with 1X PBS. Cells were incubated in ABC solution (both A and B at a dilution of 1 to 200 prepared in PBST) for 30 min, then washed 3 times with 1X PBS for 5 min each and Alexa 555 (red channel) conjugated anti biotin antibody was added to it (at a dilution of 1 to 1000 in PBST) and incubated for 1 h at room temperature. Finally, cells were washed 3 times with 1X PBS for 5 min each. The previous protocol for immunostaining with a single antibody was followed.

### TNF-Alpha ELISA

ELISA was carried out according to the manufacturer’s protocol (porcine TNF-alpha Duo Set ELISA, from R&D Systems; cat #DY 690B). Cell culture supernatants were measured in duplicates at a dilution of 1 to 10.

#### *In vitro* Myelination Assay

Neuronal cells were grown as described (see cell culture, step 7). After 1 week in culture, freshly isolated O4+ and CD11b+ cells were added to the neuronal culture at a concentration of 40,000 cells/well in oligodendrocyte medium. The medium was changed every second day with a mixture of neuronal and oligodendrocyte media (1: 1). After two and 4 weeks of co-culture cells on the coverslips were fixed and immunostaining was carried with MAP2, MOG, and CD11b specific antibodies.

#### *In vitro* Tube Formation Assay

The Matrigel was thawed on ice. 50 μl of the Matrigel was added to each well of a flat bottom 96 well plate. Plates were incubated for 30 min at 37°C to allow gel to solidify. For monitoring of tube formation cell-permeable dye viz. Calcein AM Green, Calcein Red, and Hoechst 33342 were used. Dyes were added at a final concentration of 2 μg/ml to the endothelial cells in a 6 well plates and incubated for 30 min at 37°C with 5% CO_2_ in the dark. Cells were trypsinized and centrifuged at 2,500 × *g* for 5 min and 1 ml of 1X BDM was added to the pellet. The concentration of cells was determined. Cells were diluted in 1X BDM in the presence or absence of angiogenesis inducers and inhibitors at a concentration of 2.5–3.5 × 10^5^ cells/ml and 200 μl added to each well of a 96 well plate. FGFb was added in 1X BDM with 1% FCS at concentrations of 0, 3, 30, and 300 ng/ml to induce tube formation. Suramin, an inhibitor of tube formation was added at concentrations of 0, 5, 10, and 50 μM. The cells were gently added at the selected density to the gel-coated well. The plate was incubated at 37°C, 5% CO_2_ for 12 h. Plates were analyzed by a fluorescence microscope. Images were captured in tiff format and analyzed for quantification with freely available software ImageJ distributed by National Institute of Health (NIH).

#### Neuronal Survival Assay

One-week old neurons were plated at 40,000 cells per well on poly-L Ornithine and laminin-coated glass coverslips in 24 well cell culture plates in neuronal media (see medium composition Supplementary Table 8). Different numbers of astrocytes were plated on poly-carbonated inserts (from Invitrogen cat # 141004) for cell culture of pore size 3 μm in diameter which was coated with poly-L Ornithine and laminin in astrocyte media (see Supplementary Table 7 for composition) containing HBEGF. The experimental setup was the same for all cultures. After 2 weeks, neuronal survival was determined according to the manufacturer’s instructions with the Live/Dead Viability/Cytotoxicity kit (Invitrogen, L3224).

#### Cytotoxicity Assay With AQP4 Positive NMO Serum

For immunostaining, 4 × 10^5^ cells were plated in each well of a 24 well plate with poly-L-Ornithine and laminin coated coverslips for GFAP staining. For FACS analysis, 2 × 10^5^ cell were plated in each well of coated flat bottom 96 well plates for cell viability testing. Cells were grown for 4 weeks. Medium was changed every third day. Cells were treated with different doses of heat inactivated (incubated at 56°C for 30 min) serum from NMO patients, MS patients and healthy donors. 10% of the human serum from a healthy control was added to each well as complement source. After 12 h incubation, cells on coverslips were fixed and immunostained for GFAP. Cells on 96 well plates were trypsinized and counted on FACS in 45 s window for every samples and the percent cell death was calculated.

#### Microscopic Analysis

After mounting the coverslips on the slides with ProLong^TM^ Diamond Antifade Mountant with DAPI (#P36961, Life Technologies), the slides were dried and scanned under Inverted Fluorescence Microscope, Cell Observer HS from Zeiss at 20×, 40×, or 63× magnification. Images were captured at Axio Vision software. For each staining of different cell types, 600 to 1,000 cells were counted. Blinding of the scientist who performed the counting was performed for at least one of the replicates yielding the same results.

## Results

### Isolation of Different Cell Fractions From an Adult Pig Brain

**FIGURE 1:**
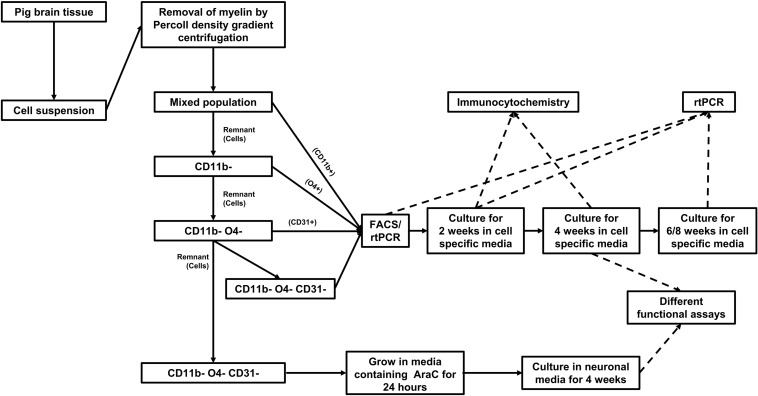
Workflow for isolation, culture, and characterization of cells isolated from adult pig brain.

Pig brain tissue was dissociated in dissociation buffer to obtain brain cell suspension. Myelin debris was removed from the suspension by Percoll gradient centrifugation. Glia subsets and endothelial cells were purified from the cell suspension in a multi-step procedure by MACS sorting (Figure 1). CD11b positive cells were first positively selected from the cell suspension. From the remaining cells O4 positive cells were positively sorted. Finally, CD31 positive cells were extracted from the remaining cell suspension. The remaining CD11b, O4, and CD31 triple negative cells were considered to contain mainly astrocytes and neurons.

**FIGURE 2:**
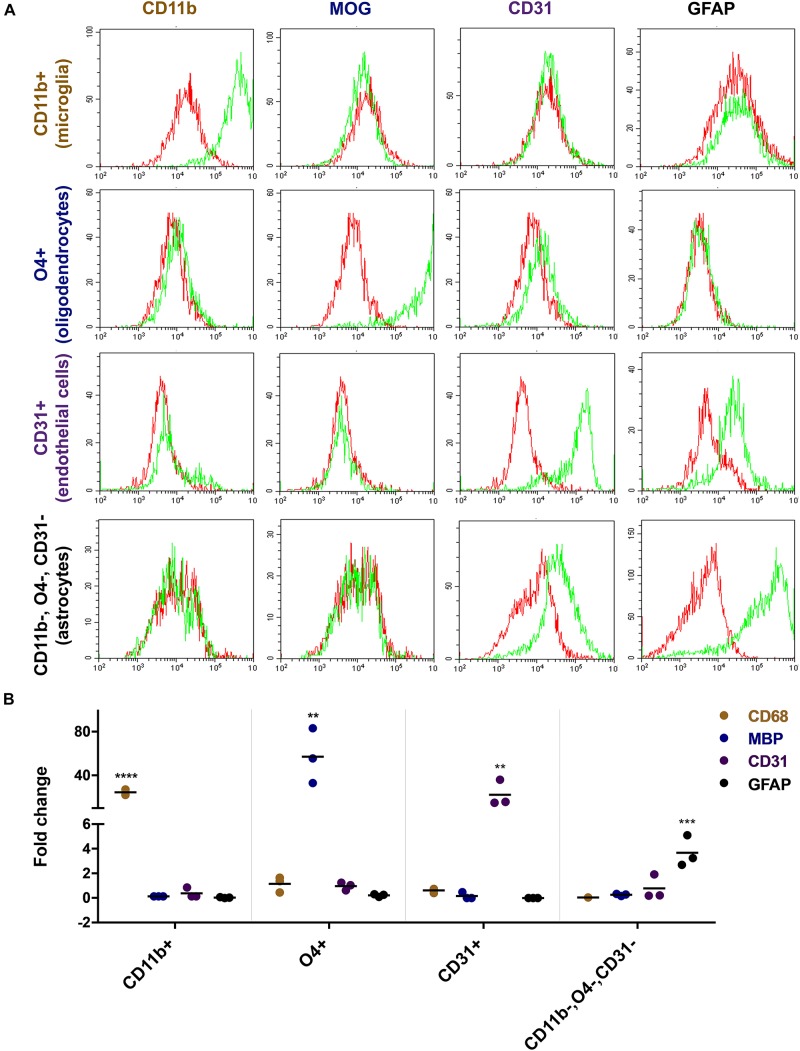
Characterization of freshly isolated glia and endothelial cells. Magnetically sorted CD11b+ (microglia), O4+ (oligodendrocytes), CD31+ (endothelial cells), and CD11b−, O4−, CD31− cells (astrocytes) were fixed and stained with lineage-specific antibodies and analyzed by flow cytometry **(A)**. Green line represents staining with the specific antibody, red line represents staining with control IgG. There is an overlap between markers related to the staining of endothelial cells and astrocytes. Both cells show to a certain extent of non-specific binding of the CD31 and GFAP antibody, respectively. RNA expression of the cells was analyzed by quantitative rtPCR analysis with probes for lineage-specific genes **(B)**. The expression level is displayed as fold expression compared to the expression level of freshly isolated pig brain cells before magnetic separation. The fold change of three independent experiments of 2–4 replicates and their medians are shown in the figure. One way ANOVA followed by Dunnett’s multiple comparisons test shows that the result is significant for each lineage-specific marker tested and shown here (^*⁣*⁣**^p < 0.0001, ^∗∗∗^*p* < 0.001, ^∗∗^*p* < 0.01).

Flow cytometric analyses with lineage-specific antibodies demonstrated the purity of the isolated cell fractions. The CD11b sorted fraction (microglia) expressed CD11b but not MOG, CD31, or GFAP and the O4 sorted fraction (oligodendrocytes) expressed MOG but not CD11b, CD31, or GFAP. CD31 sorted cells (endothelial cells) expressed CD31 but not CD11b, MOG, or GFAP and the CD11b, O4, and CD31 triple negative fraction (astrocytes) expressed GFAP but not CD11b, MOG, or CD31 (Figure 2A). Reverse transcription PCR (rtPCR) with lineage-specific probes confirmed these results. *CD68* transcripts were only upregulated in the microglia cell fraction, *MBP* only in the oligodendrocyte fraction, *PECAM1* (CD31) only in the endothelial cell fraction and *GFAP* only in the astrocyte fraction (Figure 2B).

### Primary Glia and Endothelial Cell Culture

**FIGURE 3:**
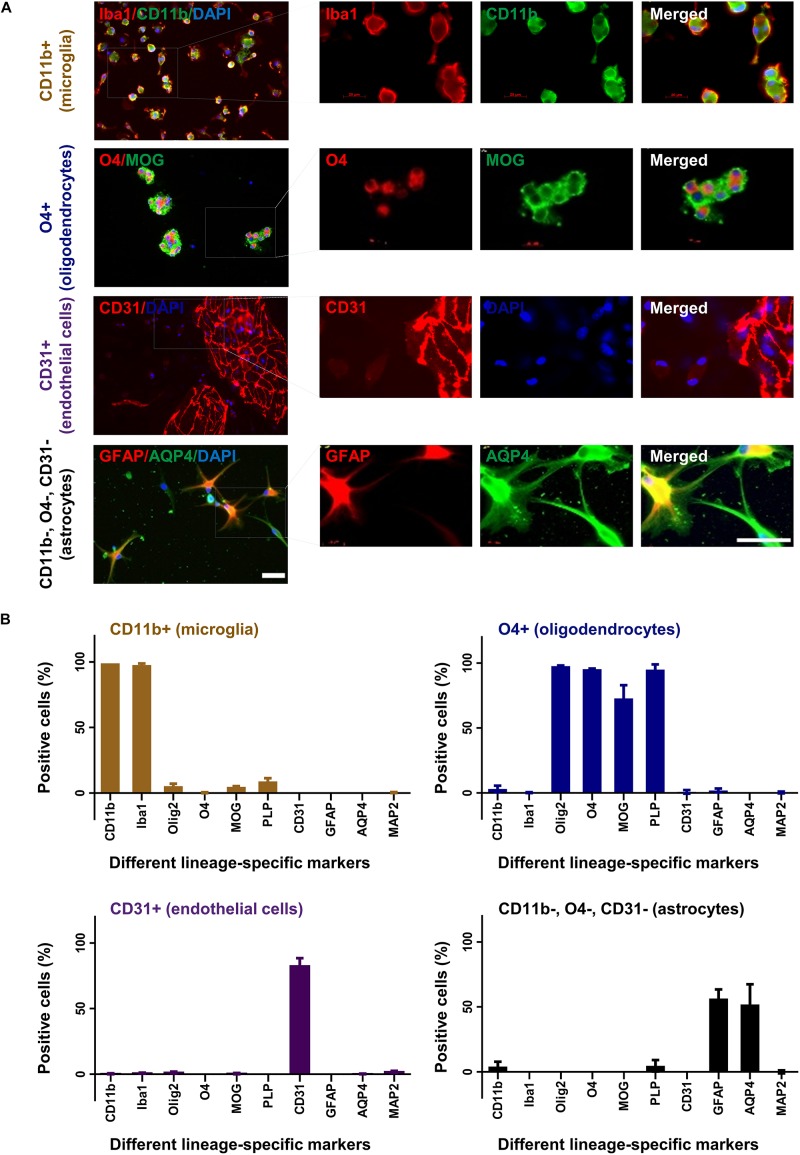
Characterization of cultured glia and endothelial cells. After 2 weeks in culture CD11b+ (microglia), O4+ (oligodendrocytes), CD31+ (endothelial cells), and CD11b–, O4–, CD31– cells (astrocytes) were fixed and stained with different lineage-specific markers **(A)**. Images were captured in 20× and 63× (insets) magnification (upper panel). Scale bars, 50 μm. Additional stainings are shown in Supplementary Figure 1. The percentage of positive cells was counted from at least three slides and displayed in the graphs **(B)**. Mean and standard deviation (as error bar) are shown in the figure. The figure is shown as a representative of three independent experiments.

The different cell fractions were cultured in specific cell culture media to support their growth and differentiation. After 2 weeks, cells were characterized by immunocytochemistry for their expression profile and purity (Figure 3A). Around 95% of cells from the CD11b sorted fraction expressed CD11b and Iba1 but were negative for O4, MAP2, CD31, PLP, GFAP, MOG, Olig2, or AQP4 compatible with a microglia phenotype (Figure 3B). Similarly, 95% of the O4 sorted cell fraction expressed the oligodendrocyte markers O4, Olig2, PLP, and MOG but no other marker. In the CD31 sorted fraction 80% of cells still expressed CD31 but were negative for all other markers. Noticeably, 1–2% of CD31+ cells were seen to be MAP2 and Olig2 positive. Finally, 60% cells from the negatively selected fraction (CD11b, O4, and CD31 triple negative) grown in astrocyte medium expressed the astrocyte markers GFAP and AQP4.

**FIGURE 4:**
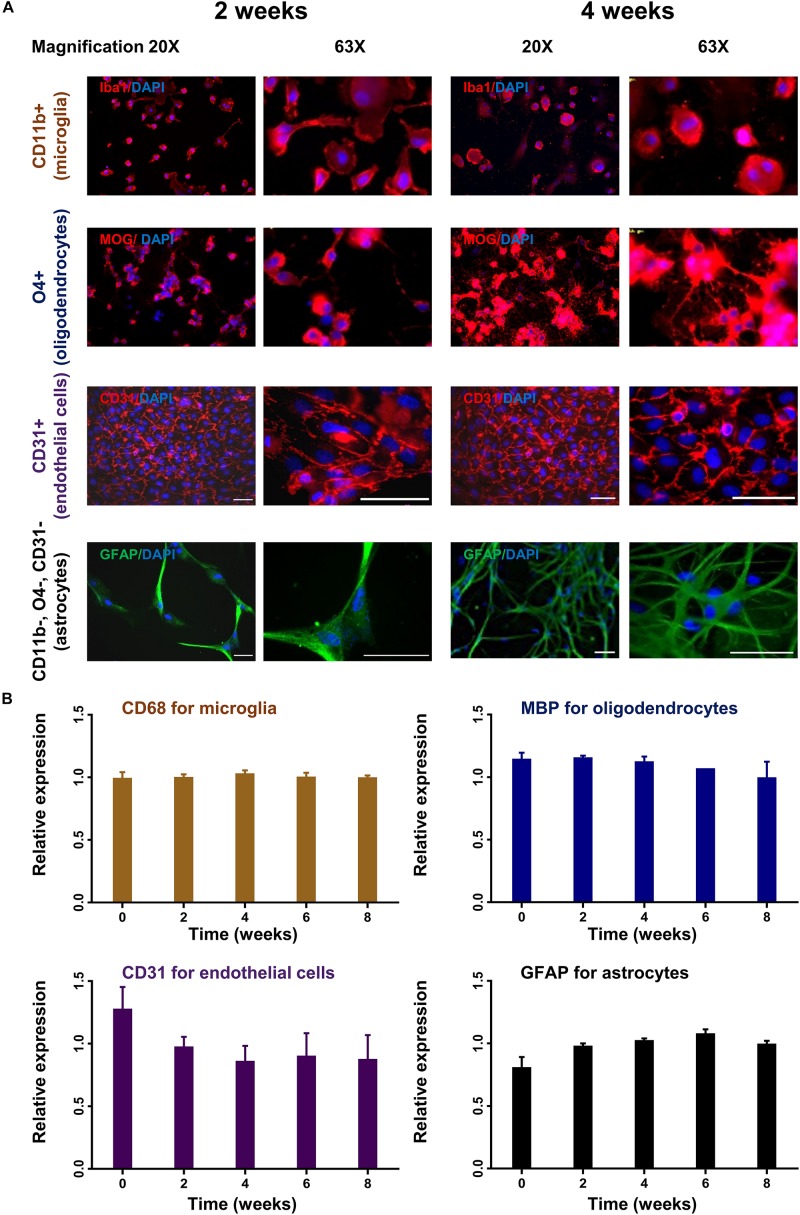
Time course of lineage-specific marker expression in cultured glial cells. CD11b+ (microglia), O4+ (oligodendrocytes), CD31+ (endothelial cells), and CD11b–, O4–, CD31– cells (astrocytes) were cultured for up to 8 weeks. Cells were analyzed for morphological changes and expression of lineage-specific markers by immunohistochemistry **(A)**. Images were captured in 20× and 63× magnification. Scale bars, 50 μm. Cells were also analyzed by quantitative rtPCR with probes for lineage-specific genes. Relative expression compared to the expression of endogenous gene (*Rpl13a*) is shown **(B)**. Mean and standard deviation (as error bar) are shown in the figure. The result is a representation of three independent experiments. One way ANOVA followed by Dunnett’s multiple comparisons test shows that the ratio of expression of the lineage-specific markers and housekeeping gene (*Rpl13a*) is not significantly different at week 2–8 compare to baseline (ns).

To monitor changes in morphology and expression profile, different cell subsets were cultured for up to 8 weeks and analyzed by immunocytochemistry and rtPCR. Oligodendrocytes and astrocytes showed increasing cell processes and branching during the observation period in culture reflecting cellular differentiation (Figure 4A). Microglia and endothelial cells did not show significant changes in their morphology during culture (Figure 4A). Only endothelial cells were subcultured with 1:4 ratio. The stable expression of lineage-specific markers during culture was confirmed by rtPCR. The expression of *MBP* for oligodendrocytes, *GFAP* for astrocytes, *CD68* for microglia and *PECAM1* (CD31) for endothelial cells remained rather stable up to 8 weeks in culture (Figure 4B).

### Functional Characterization of Cultured Cells

**FIGURE 5:**
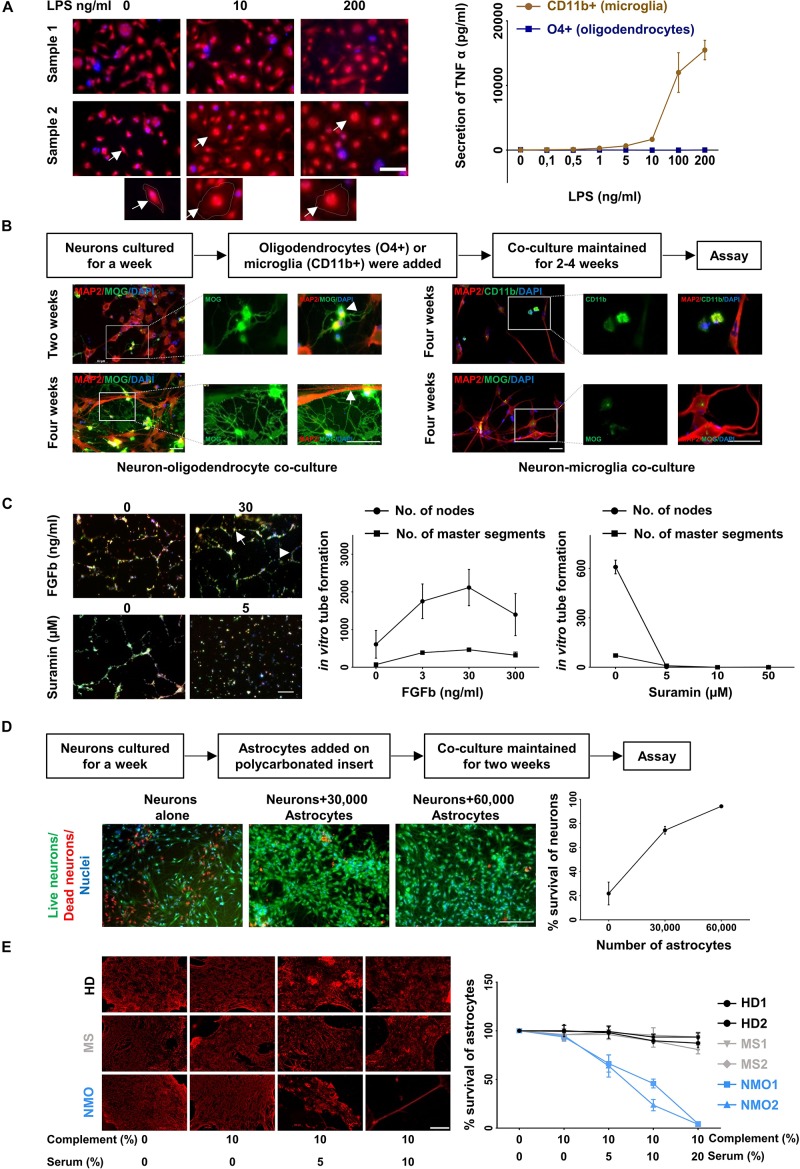
Functional characterization of cultured glia and endothelial cells. **(A)** LPS induced activation of microglia (CD11b+ cell fraction) was determined by morphological changes after staining with Calcein Red- Orange and DAPI (left panel) and the release of TNF-alpha (right panel). Scale bars, 50 μm. One cell from each condition is marked by an arrow and shown at a higher resolution at the bottom of the figure. Oligodendrocytes (O4+ cell fraction) served as a control for the TNF-alpha release assay. The result is a representation of three independent experiments. Microglia respond to LPS significantly more than oligodendrocytes (*p* < 0.0001 at 200 ng/ml of LPS treatment, Mann–Whitney test). **(B)** Oligodendrocytes (O4+ fraction, left panel) were co-cultured with neurons generated from the triple negative cell fraction (CD11b−, O4−, and CD31−). Neuronal cultures with microglia served as control (right panel). The cells were fixed and stained with MAP2, MOG, and CD11b. A typical branched morphology of oligodendrocyte is shown by an arrowhead. Wrapping of axons by oligodendrocytes is indicated by a white arrow. Scale bars, 50 μm. A representative result from three independent experiments is shown here. **(C)** Fluorescence labeled primary endothelial cells generated from CD31 sorted cells were plated in the presence of different concentration of FGFb and different concentration of Suramin. After 6 h cells were analyzed for master segments and branch formation by fluorescence microscopy (left panel). Scale bars, 200 μm. A representative node and a master segment are shown by an arrow and an arrowhead, respectively. Images were analyzed with ImageJ software for the presence of number of nodes and number of master segment (right panel). The figure is the representative image of three independent experiments. The result shows that the node formation is significantly increased in response to 30 ng/ml of FGFb whereas it is decreased substantially in response to the lowest dose of Suramin 5 μgm/ml. **(D)** One-week old neurons generated from triple negative cells (CD11b–, O4–, and CD31–) as mentioned in the method section were plated and astrocytes on a polycarbonate insert were added. The co-culture was maintained for 2 weeks (left panel). Cells were stained with live/dead dyes where live cells are stained green (Calcein) and dead cells are stained red (EtBR). Cells were counterstained with Hoechst 3342 for nuclear staining. Scale bars, 200 μm. The percentage (%) of living cells was calculated and plotted against the number of astrocytes (right panel). The result is a representation of three independent experiments. One way ANOVA followed by Dunnett’s multiple comparisons test shows that neuronal survival is increased significantly on the addition of both of 30,000 and 60,000 astrocytes (*p* < 0.001). **(E)** Astrocytes cultured from triple negative cells (CD11b–, O4–, and CD31–) were exposed to different concentration of heat inactivated AQP4 antibody positive sera from patients with NMO patients. Sera from patients with multiple sclerosis (MS) patients and healthy donors (HD) served as controls. All sera were supplemented with 10% human serum from a healthy donor as complement source. After 12 h cells were fixed and stained for GFAP (left panel). Scale bars, 100 μm. Similarly, after 12 h of treatment the cells were trypsinized and the number of living cells counted by flow cytometry. The percentage of live cells was plotted against the concentration of serum (right panel). Mean and standard deviation (as error bars) are shown in the graph. The result is the representation of three independent experiments. Killing of astrocytes is significantly higher with NMO sera than others (*p* < 0.0001, Kruskal–Wallis test at 20% of serum treatment).

Next, we examined the functionality of the cultured cells. CD11b positive cultured cells were treated with different concentration (0.1 to 200 ng/ml) of lipopolysaccharides (LPS) for 4 h and analyzed for morphological changes. Upon stimulation with higher concentration of LPS the cells developed a amoeboid shape characteristic for an activated phenotype (Figure 5A, left panel). Treatment of these cells with LPS resulted in a dose dependent release of tumor necrosis factor alpha, a characteristic feature of microglia cells (Figure 5A, right panel).

O4 positive cells were co-cultured with neurons, which were generated from the CD11b, O4, and CD31 triple-negative cell fraction cultured in neuronal medium. Immunostaining with the neuronal marker MAP2 and myelin-specific antibody against MOG revealed myelination of neurons in the co-culture condition (Figure 5B, lower left panel). After 2 weeks oligodendrocytes started to show typical branched oligodendrocyte morphology (arrowhead) (Figure 5B, upper left panel) and after 4 weeks wrapping of axons (Figure 5B, lower left panel). No myelination was found when neuronal cells were cultured with microglia (Figure 5B, right panel).

One of the characteristic features of endothelial cells is their ability to form a tube-like structure while grown on a Matrigel. Fibroblast growth factor – basic (FGFb) is a potent inducer of this process (Bikfalvi et al., 1997) and Suramin reduces the tube formation drastically acting as an inhibitor (Meyers et al., 2000). Fluorescence labeled endothelial cells (passage 3–6, 3–4 weeks in culture) were sub-cultured in the presence of FGFb and Suramin over a Matrigel for 6 h. We observed *in vitro* tube formation with increasing concentration of FGFb, which was blocked by Suramin even at the lowest concentration used (Figure 5C).

It is well established that astrocytes promote neuronal survival when co-cultured with neurons (Banker, 1980; Zhang et al., 2016). One-week old neurons were plated on coated coverslips and different numbers of astrocytes on a poly carbonated insert were added over it. The co-culture was maintained for 2 weeks. Neuronal survival was enhanced with increasing numbers of astrocytes added. Neurons cultured without astrocytes served as control (Figure 5D).

Astrocyte cultures were exposed to Aquaporin-4 (AQP4) antibody containing sera from patients with Neuromyelitis optica (NMO), a disease characterized by specific loss of astrocytes (Lennon et al., 2005; Misu et al., 2007). In the presence of complement, AQP4 specific antibodies are cytotoxic for AQP4 positive astrocytes *in- vivo* and *vitro* (Bennett et al., 2009; Bradl et al., 2009). Astrocytes were cultured for 4 weeks and then exposed to different concentrations of APQ4 antibody containing sera from NMO patients. Sera from other patients not suffering from NMO served as control. After 12 h of incubation, the cells were fixed and stained for GFAP. A dose dependent loss of GFAP positive cells in the presence of NMO but not control sera was observed (Figure 5E, left panel). In parallel the number of live cells after exposure to NMO and control sera was determined by flow cytometry. NMO sera but not control sera induced a dose dependent loss of astrocytes *in vitro* (Figure 5E, right panel).

## Discussion

Primary glial and endothelial cell cultures have been beneficial to study the function and dysfunction of these cells *in vitro*. Most methods of cell cultures are generated from rodent brain tissue although it has become more and more evident that rodent glia and endothelial cells substantially differ from the human counterpart. The differences include morphological, transcriptomic and proteomic differences (Lynch and Conery, 2003; Stellwag, 2004; Frith et al., 2005; Gustincich et al., 2006; Koonin, 2009; Markov et al., 2010; Schad et al., 2011; Ho et al., 2016). Thus, rodent primary cell cultures might not be ideally suited to study glial or endothelial dysfunction related to human diseases. We decided to develop a method to establish primary glia and endothelial cell culture from pig brain. Pigs share more than 90% of genetic elements with humans (Swindle et al., 2012; Yu et al., 2015). The overall macroscopic structure, i.e., size and weight (Sauleau et al., 2009; Gieling et al., 2011; Roura et al., 2016) and microscopic structure, i.e., white/gray matter ratio, gyral complexity, etc. (Lind et al., 2007; Sauleau et al., 2009; Howells et al., 2010) of pig brain is quite similar to that of human brain. Easy availability of pig brain tissue and the large size of the brain allow isolating a large number of cells from a single brain. We established a method that enables to generate primary microglia, oligodendrocyte, endothelial cell and astrocytes cultures in parallel from the adult pig brain. Primary cells cultures reached a high purity and stable phenotype over 8 weeks of culture. Functional assays confirmed the efficacy of cultured glial and endothelial cells.

Previous studies have reported protocols for purification and culture of mature oligodendrocytes from adult pig (Althaus et al., 1984; Gebicke-Harter et al., 1984a, b). Similar methodologies were used to isolate and culture pig neural progenitor cells (Rasmussen et al., 2013), astrocytes (Bobilya, 2012), and microglia (Ionescu et al., 2011). Recently a protocol was reported to isolate and culture endothelial cells from pig brain microvessels (Patabendige and Abbott, 2014). Some of these methods involve Percoll density centrifugation for isolation of specific cells, which does not provide very high purity. Other methods involved culture of the cells before purification, which does not allow to analyze freshly isolated cells. Moreover, none of the reported methods allows to purify all different glia and endothelial cells from an adult pig in parallel and gives access to freshly isolated as well as cultured cells.

Generation and maintenance of human induced pluripotent stem cells (iPSCs) is an alternative strategy to study the mechanism and pathophysiology of many diseases associated with glial cells (Zheng et al., 2018) or endothelial cells (Luo et al., 2017). It has also been shown that human iPSCs can be differentiated to endothelial cells (Luo et al., 2017). Human iPSCs technology shows a massive prospect in personalized medicine. However, our method is easier and less expensive than iPSC technology because, (i) reprogramming of cells to iPSCs is not trivial and required high expertise, (ii) it is an expensive method, (iii) takes a lot of time, and (iv) differentiating iPS cells into three/four different mature cell types simultaneously is not an easy process. Unlike all methods for isolation and culturing of glial and endothelial cells reported so far, we were able to isolate and culture different types of glial cells and endothelial cells from a single pig brain in parallel. Cells were maintained in culture for up to 8 weeks without significant changes. The functional integrity of the cells was proven by different cell type-specific assays. Glia and endothelial cells were prepared in bulk, which allows to set up co-culture experiments and investigate different glia and endothelial cells in parallel. We believe that our differentiation protocol starting from a broad population of freshly isolated cells is of advantage because it may better reflect the polyclonal nature of glial cells in the brain. Because there is much more knowledge at the molecular level (e.g., transcriptomics and proteomics) in human and rodent cells, our method is complementary to current iPS cells and primary glia cultures from rodents. In summary, we provide an inexpensive and comprehensive method for primary glia and endothelial cell culture, which might be very useful in translational research for human brain diseases.

## Data Availability

All datasets generated for this study are included in the manuscript and/or the Supplementary Files.

## Author Contributions

GKT performed the experiments. BH supervised the study. GKT and BH analyzed the data and wrote the manuscript. RS and SRK helped in the experiments and analysis. CN helped in some of the experiments. All authors read and approved the manuscript.

## Conflict of Interest Statement

During the last 2 years, BH has served on Scientific Advisory Boards for Novartis. He has served as DMSC member for AllergyCare and TG Therapeutics. He or his institution have received speaker honoraria from Desitin. Holds part of two patents – one for the detection of antibodies against KIR4.1 in a subpopulation of MS patients and one for genetic determinants of neutralizing antibodies to interferon β.

The remaining authors declare that the research was conducted in the absence of any commercial or financial relationships that could be construed as a potential conflict of interest.
