# Supplementary material for: Isolation, Culture and Functional Characterization of Glia and Endothelial Cells From Adult Pig Brain
Source: Front Cell Neurosci. 2019 Jul 23;13:333. doi: 10.3389/fncel.2019.00333 (PMC6705213; doi:10.3389/fncel.2019.00333)
Supplement: Supplementary file 1 [file Table_1.docx]

**Isolation, culture and functional characterization of glia and endothelial cells from adult pig brain**

Goutam Kumar Tanti^1^, Rajneesh Srivastava^1^, Sudhakar Reddy Kalluri^1^, Carina Nowak^1^ and Bernhard Hemmer*^1,2^

^1^Department of Neurology, School of Medicine, Technical University of Munich, Munich Germany

^2^Munich Cluster for Systems Neurology (SyNergy), Munich, Germany.

Correspondence:

Bernhard Hemmer

Department of Neurology,

Klinikum rechts der Isar, Technical University of Munich

Ismaninger Str. 22

81675 Munich

Germany

Phone +498941404601

Fax +498941407681

Email: hemmer@tum.de

**Supplementary material**

**Supplemental table 1: Components of the dissociation buffer**

| S No | Components | Company (Catalogue Number) | Stock Concentration | Final Concentration |
| --- | --- | --- | --- | --- |
| 1 | HBSS | Thermo Fisher Scientific (#14025092) | To make up the volume | |
| 2 | D (+) Glucose | Sigma (#G8270) | 2.25 M | 22.5 mM |
| 3 | Fructose | Sigma (#F3510 | 2.25 M | 22.5 mM |
| 4 | NaHCO_3_ | Sigma (#S5761 | 1 M | 26 mM |
| 5 | DNase I | Sigma (#D-4527 | 10 mg/ml | 1 mg/ml |
| 6 | Collagenase D | Worthington (#LS004197) | 50 mg/ml | 5 mg/ml |
| 7 | Hyaluronidase | Worthington (#LS005474) | 5 k Unit/ml | 10 unit/ml |
| 8 | L-cysteine | Sigma (#W326305) | 1 M | 1 mM |
| 9 | CaCl_2_ | Sigma (#C1016) | 1 M | 3 mM |
| 10 | KH_2_PO4 | Sigma (#P5655) | 1 M | 25 mM |
| 11 | Pen/Strep | Gibco (#15070) | 1 % | 1% |

**Supplemental table 2: Components of the Basal Chemically Defined Media (BDM)**

| S No | Components | Company (Catalogue Number) | Stock Concentration | Final Concentration |
| --- | --- | --- | --- | --- |
| 1 | L Glutamine | Gibco (#00473) | 100X (200mM) | 1X (2mM) |
| 2 | Sodium Pyruvate | Sigma (#P2256) | 100 mM | 1 mM |
| 3 | Transferrin | Sigma (#T8158) | 50 mg/ml | 50 µg/ml |
| 4 | BSA | Sigma (#A9647) | (10% in DMEM) | 0.1 % |
| 5 | Insulin) | Sigma (#I6634 |  | 5 µg/ml |
| 6 | - Hydrocortisone | Sigma (#H0888) | 10 µM | 10 nM |
| 7 | Sodium Selenite | Sigma (#S5261) | 30 µM | 30 nM |
| 8 | - D-biotin | Sigma (#B4501) | 10 µM | 10 nM |
| 9 | Progesterone | Sigma (#P8783) | 30 mg/ml | 60 ng/ml |
| 10 | DMEM | Gibco (#11960-044) | To make up the volume | |

**Supplemental table 3: Reagents for cell separation and culture**

| S No |  | Company (Catalogue No) | Dilution |
| --- | --- | --- | --- |
| 1 | CD11b | MACS (#5180516447) | 1:10 in separation buffer |
| 2 | O4 | MACS (#5180522145) | 1:10 in separation buffer |
| 3 | CD31 | MACS (#5180523016) | 1:10 in separation buffer |
| 4 | FcRn blocker | MACS (#5180219621) | 1:10 in separation buffer |
| 5 | L-ornithine | Sigma (#P3655) | 20 µg/ml |
| 6 | Laminin | Life Technologies (#23017015) | 1 µg/ml |
| 7 | Cover slips  (of 12mm diameter number 1 glass) | Thermo Fisher Scientific (#LOT 0288) | 1 µg/ml |
| 8 | Hoechst 33342 | Thermo Fisher Scientific (#H1399) | 2 µg/ml |
| 9 | CellTrace™ Calcein Green, AM | Thermo Fisher Scientific (#C34852) | 2 µg/ml |
| 10 | CellTrace™ Calcein Red, AM | Thermo Fisher Scientific (#C34851) | 2 µg/ml |
| 11 | Matrigel Matrix | Corning (#356237) | N/A |

**Supplemental table 4: Microglia growth medium**

| S No | Components | Company (Catalogue Number) | | Stock Concentration | Final Concentration |
| --- | --- | --- | --- | --- | --- |
| 1 | BDM |  | | 10X | 1X |
| 2 | FCS | (Sigma #F7524) | | 100% | 10% |
| 3 | Pen/Strep | (Thermo Fisher Scientific #15070) | | 100% | 1% |
| 4 | DMEM | Gibco (#11960-044) | To make up the volume | | |

**Supplemental table 5: Oligodendrocyte medium**

| S No | Components | Company (Catalogue Number) | Stock Concentration | Final Concentration |
| --- | --- | --- | --- | --- |
| 1 | BDM |  | 10X | 1X |
| 2 | NAC | Sigma (#A7250) | 1000X | 1X |
| 3 | T3 | Sigma (#T2752) | (0.5 mM) | 1X |
| 4 | T4; (L-Thyroxine) | Sigma (#T2376) | 1000X | 1X |
| 5 | CNTF | Sigma (#C3710) | 50 µg/ml | 10 ng/ ml |
| 6 | Pen/Strep | Thermo Fisher Scientific (#15070) | 100X | 1X |
| 7 | PDGF | Sigma (#H8291) | 50 µg/ml | 10 ng/ml |
| 8 | Heat Inactivated HS | Sigma (#H3667) | 100% | 0.5 % |
| 9 | DMEM | Gibco (#11960-044) | To make up the volume | |

**Supplemental table 6: Endothelial growth medium**

| S No | Components | Company (Catalogue Number) | Stock Concentration | Final Concentration |
| --- | --- | --- | --- | --- |
| 1 | BDM |  | 10X | 1X |
| 2 | Endothelial Supplement | Promocell (#C-39225) | 100% | 5% |
| 3 | Pen/Strep | Thermo Fisher Scientific (#15070) | 100% | 1% |
| 4 | DMEM | Gibco (#11960-044) | To make up the volume | |

**Supplemental table 7: Astrocyte medium**

| S No | Components | Company (Catalogue Number) | Stock Concentration | Final Concentration |
| --- | --- | --- | --- | --- |
| 1 | BDM |  | 10X | 1X |
| 2 | Pen/Strep | Thermo Fisher Scientific (#15070) | 100 | 1% |
| 3 | FCS | (Sigma #F7524) | 100% | 1% |
| 4 | Heparin-Binding Epidermal Growth Factor (HBEGF) | Sigma (#E4643) | 5 µg/ml | 5 ng/ml |
| 5 | DMEM | Gibco (#11960-044) | To make up the volume | |

**Supplemental table 8: Neuronal medium**

| S No | Components | Company (Catalogue Number) | Stock Concentration | Final Concentration |
| --- | --- | --- | --- | --- |
| 1 | Neurobasal Medium | Gibco (#21103-049) | To make up the volume | |
| 2 | N2 Supplement | Gibco (#A13707-01) | 100X | 1X |
| 3 | dBcAMP | Sigma (#D0627) | 0.25M | 0.25 mM |
| 4 | Pen/Strep | Thermo Fisher Scientific (#15070) | 100X | 1X |
| 5 | Glutamax | Gibco (#00473) | 100X | 1X |

**Supplemental table 9: Taqman probes**

| S No | Gene | Company (Catalogue Number) | Assay |
| --- | --- | --- | --- |
| 1 | *CD68* | Thermo Fisher Scientific (#4448514) | Customized CD68_NM_00129177 |
| 2 | *MBP* | Thermo Fisher Scientific (#4331182) | Ss03385047_u1 |
| 3 | *PECAM1* (31) | Thermo Fisher Scientific (#4331182) | Ss03392600_u1 |
| 4 | *GFAP* | Thermo Fisher Scientific (#4331182) | Ss03373545_g1 |
| 5 | *Rpl13a* | Thermo Fisher Scientific (#4351372) | Ss03376908_u1 |

**Supplemental table 10: Antibody details and their dilutions used**

| S No | Antibody | Company (Catalogue Number) | Host | Dilution used | |
| --- | --- | --- | --- | --- | --- |
|  |  |  |  | Immunocytochemistry | FACS |
| 1 | Olig2 | ABCam: (#ab136253) | Rabbit polyclonal | 1:700 in 5% Goat serum |  |
| 2 | MAP2 | Millipore (#AB5622) | Rabbit polyclonal | 1:1000 in 5% Goat serum |  |
| 3 | GFAP | Life Technologies (#13-0300) | Rat monoclonal IgG | 1:200 in 5% Goat serum | 1:50 in FACS Buffer |
| 4 | CD11b | Antigenix America (#APG112) | Mouse monoclonal hybridoma | 1:10 in PBST (PBS with 0.1% Tween 20) | 1:50 in FACS Buffer |
| 5 | O4 | Millipore (#MAB345) | Mouse monoclonal IgM | 1:25 in PBST |  |
| 6 | MOG | 8-18C  in house | Mouse Hybridoma | 1:2 in 5% Goat serum | 1:10 in FACS Buffer |
| 7 | AQP4 | Sigma  (#A5971) | Rabbit polyclonal | 1:200 in 5% Goat serum |  |
| 8 | Iba1 | AbCam  (#Ab5076) | Goat polyclonal | 1:200 in 5% Rabbit serum |  |
| 9 | CD31 | BioRad  (#MCA1746GA) | Mouse monoclonal IgG | 1:200 in 5% Goat serum | 1:50 in FACS Buffer |

Figure Legends:

Supplementary Figure 1: After two weeks in culture CD11b+ (microglia), O4+ (oligodendrocytes), CD31+ (endothelial cells) and CD11b-, O4-, CD31- cells (astrocytes) were fixed and stained with different lineage-specific markers (A). Images were captured in 20X magnification. Scale bars, 50 μm.
