# Supplementary figures and images for: Isolation, Culture and Functional Characterization of Glia and Endothelial Cells From Adult Pig Brain
Source: Front Cell Neurosci. 2019 Jul 23;13:333. doi: 10.3389/fncel.2019.00333 (PMC6705213; doi:10.3389/fncel.2019.00333)

Supplementary Figure 1

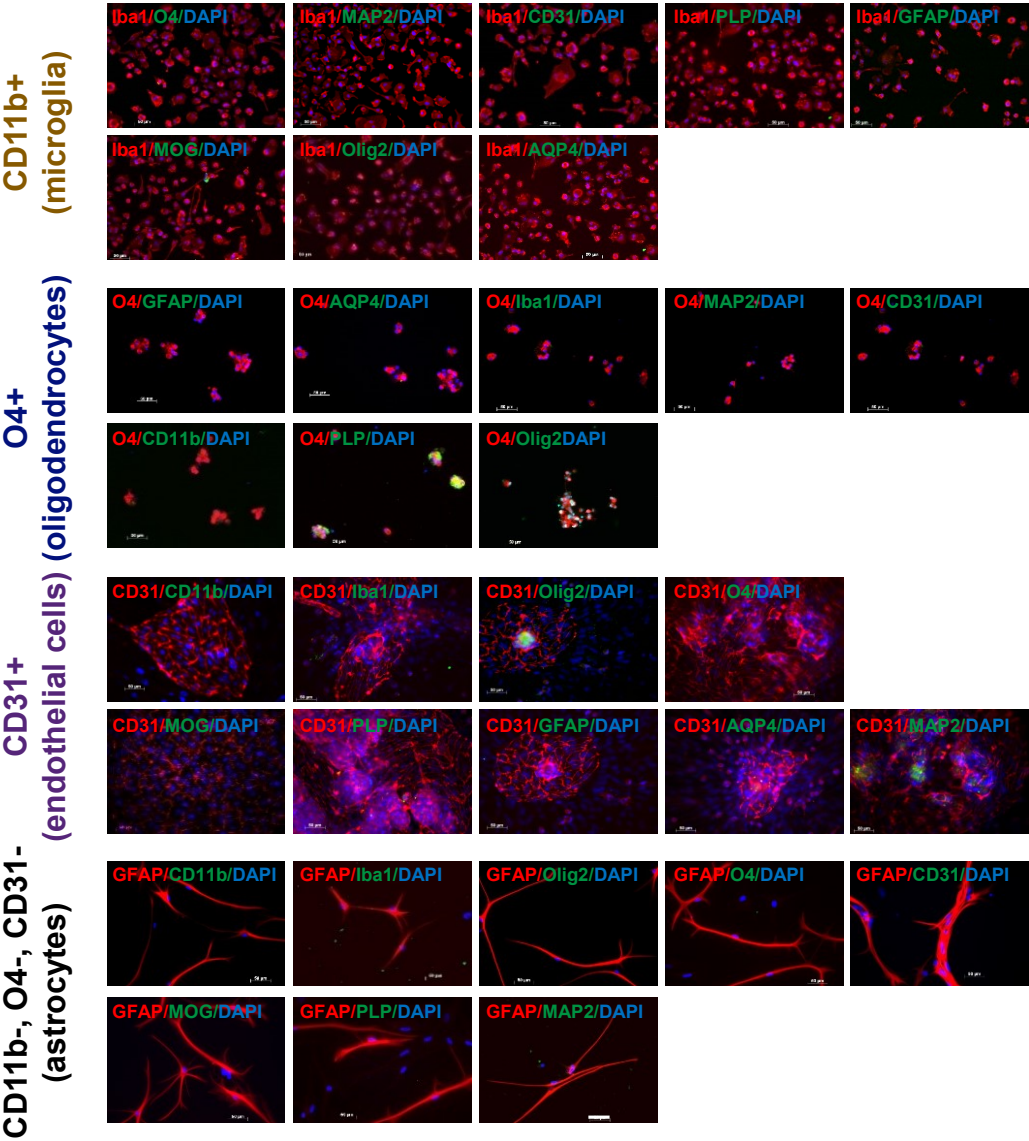

Supplement: Supplementary file 2 [file Data_Sheet_1.PDF]
